# Salvianolic acids and its potential for cardio-protection against myocardial ischemic reperfusion injury in diabetes

**DOI:** 10.3389/fendo.2023.1322474

**Published:** 2024-01-12

**Authors:** Yuxin Jiang, Yin Cai, Ronghui Han, Youhua Xu, Zhengyuan Xia, Weiyi Xia

**Affiliations:** ^1^ Department of Anesthesiology, Affiliated Hospital of Guangdong Medical University, Guangdong, China; ^2^ Department of Health Technology and Informatics, The Hong Kong Polytechnic University, Hong Kong, Hong Kong SAR, China; ^3^ Faculty of Chinese Medicine State Key Laboratory of Quality Research in Chinese Medicine, Macau University of Science and Technology, Avenida Wai Long, Taipa, Macao SAR, China; ^4^ State Key Laboratory of Pharmaceutical Biotechnology, Department of Medicine, The University of Hong Kong, Hong Kong, Hong Kong SAR, China; ^5^ Doctoral Training Platform for Research and Translation, BoShiWan, GuanChong Village, Shuanghe Town, ZhongXiang City, Hubei, China

**Keywords:** salvianolic acids A, salvianolic acids B, myocardial ischemia reperfusion injury, diabetics introduction, cardioprotection

## Abstract

The incidence of diabetes and related mortality rate increase yearly in modern cities. Additionally, elevated glucose levels can result in an increase of reactive oxygen species (ROS), ferroptosis, and the disruption of protective pathways in the heart. These factors collectively heighten the vulnerability of diabetic individuals to myocardial ischemia. Reperfusion therapies have been effectively used in clinical practice. There are limitations to the current clinical methods used to treat myocardial ischemia-reperfusion injury. As a result, reducing post-treatment ischemia/reperfusion injury remains a challenge. Therefore, efforts are underway to provide more efficient therapy. Salvia miltiorrhiza Bunge (Danshen) has been used for centuries in ancient China to treat cardiovascular diseases (CVD) with rare side effects. Salvianolic acid is a water-soluble phenolic compound with potent antioxidant properties and has the greatest hydrophilic property in Danshen. It has recently been discovered that salvianolic acids A (SAA) and B (SAB) are capable of inhibiting apoptosis by targeting the JNK/Akt pathway and the NF-κB pathway, respectively. This review delves into the most recent discoveries regarding the therapeutic and cardioprotective benefits of salvianolic acid for individuals with diabetes. Salvianolic acid shows great potential in myocardial protection in diabetes mellitus. A thorough understanding of the protective mechanism of salvianolic acid could expand its potential uses in developing medicines for treating diabetes mellitus related myocardial ischemia-reperfusion.

## 1 Introduction

Myocardial ischemia is one of the most common types of cardiovascular disease that increases morbidity and mortality worldwide (1). Effective limitation of infarct size through timely restoration of blood flow to ischemic myocardium is the standard treatment to rescue ischemic myocardium and thus to improve the patient outcomes. Paradoxically, reperfusion itself causes cardiac injury, which is known as myocardial ischemia/reperfusion injury (MI/RI). Moreover, patients with diabetes are more vulnerable to MI/RI than those without diabetes (2), yet the underlying mechanism is incompletely understood. The complicities of the MI/RI which includes oxidative stress, calcium overload, inflammatory response, energy metabolism disorder, mitochondrial dysfunction, and apoptosis were shown in many studies (3, 4). Oxidative stress is known as an essential factor in myocardial ischemia reperfusion (I/R) (5, 6), and oxidative stress levels in the myocardium of diabetic patients were found to be significantly higher than that in non-diabetes. This could be one of the mechanisms attributable to the increased myocardial vulnerability to MI/RI in diabetes. In addition to increases in reactive oxygen species (ROS) and oxidative stress, increases in inflammation, reduction in cardiac Akt and STAT3 all occur in the myocardium of diabetes (7). These elements play important roles in diabetics complicated by MI/RI and may be attributed to the increased myocardial sensitivity to MI/RI (8, 9).

Danshen (Salvia miltiorrhiza Bunge), a traditional Chinese medicine that has been widely prescribed to patients with angina pectoris and hyperlipidemia was found to have a preventive effect in type 2 diabetic patients and in type 2 diabetic rats with nephropathy (10, 11). The chemical constituents of Danshen can be classified into two categories: water-soluble (hydrophilic) phenolic compounds and nonpolar (lipophilic) diterpenoidal compounds. Salvianolic acids are the major hydrophilic constituents amongst all. Among salvianolic acids, salvianolic acid A (SAA), salvianolic acid B (SAB), rosmarinus acid, danshensu, caffeic acid, and lithospermic acid are the main phenolic acids. SAA and SAB, in particular, are polyphenolic compounds known to have powerful antioxidant capacities (12).

Recent studies have demonstrated that SAA can exert anti-diabetic effects, preventing diabetic complications by reducing inflammatory response and improving lipid disorders (13), revealing the possible therapeutic effect of SAA on DM (14). Diabetes with MI/RI are not sensitive to pre-, post-conditioning cardioprotective interventions that are otherwise effective in non-diabetic subjects, while the related mechanisms are unclear. SAA can alleviate diabetes complications like vascular disease (14), but few studies support SAA can reduce MI/RI in DM and the mechanism has not been explored.

This review aims to provide a collective understanding of the potential effect of salvianolic acids in protecting against diabetes and myocardial ischemia-reperfusion in recent years and to explore whether salvianolic acid has the potential protective effects in Diabetes that are complicated by MI/RI. It is hopeful that such a collective understanding will help develop new therapeutic interventions for the clinical treatment of diabetic myocardial ischemia-reperfusion.

## 2 The pathogenesis and mechanism of MIRI

In 1960, Jennings et al. first reported MIRI, which is a condition that occurs when there is a temporary interruption of blood flow to the heart (ischemia) followed by the restoration of blood flow (reperfusion) (15). Restoring blood flow, such as percutaneous coronary intervention (PCI) or coronary artery bypass grafting (CABG) is the most effective method to improve patient outcome (16). However, reperfusion itself causes damage to the myocardium, leading to exacerbation of the initial ischemic injury, and as such, even effective restoration of blood flow does not attenuate MIRI (4). Reperfusion triggers a series of tissue responses that contribute to the injury. This includes the production of oxygen free radical and mitochondrial damage, release of inflammatory factors, endoplasmic reticulum stress, and amplification of tissue damage (17). MIRI results from complex pathophysiological mechanisms, including oxidative stress, inflammatory response, endothelial cell dysfunction, mitochondrial dysfunction, calcium overload, apoptosis and autophagy (18).

### 2.1 Oxidative stress

Reactive oxygen species (ROS) are small reactive molecules that play a significant role in various cellular functions and biological processes, including cell signaling and homeostasis, in almost all eukaryotic cells. Some examples of ROS include superoxide anion radical, hydrogen peroxide (H_2_O_2_), hydroxyl radical (OH^-^), ozone (O_3_), and singlet oxygen (O_2_). (19). Under physiological conditions, ROS production is tightly regulated and plays a beneficial role in cell proliferation and metabolism (20). However, when ROS levels becomes excessively high, they can cause oxidative stress, which is a state of imbalance between the production of ROS and the ability of cells to detoxify them or repair the resulting damage (21). Overexpression of ROS and hypoxia in the tissue microenvironment can disrupt normal tissue repair and regeneration. This disruption can contribute to the development of fibrosis, dysfunction, and severity of cardiovascular diseases.

The accumulation of ROS during I/R injury is a major cause of oxidative damage. This phenomenon is also observed in diabetic myocardial injury (22). A study by Liu et al. revealed that I/R-induced apoptosis is mainly a consequence of excessive oxidative stress. Persistent cellular injury including necrosis and apoptosis of cardiomyocytes are the result of intense oxidative stress, which in turn trigger mitochondrial production of ROS in the early stages of ischemia in response to many mildly harmful stimuli to modulate stimulus-induced tolerance to ischemia. During reperfusion, the electron transport chain dysfunction, specifically the dysfunction in complex I (NADH dehydrogenase) and III (coenzyme Q-cytochrome c reductase), leads to excessive release of ROS. This results in the production of superoxide anions (O2-·), which are converted to hydrogen peroxide (H2O2) by the action of superoxide dismutase. In the presence of Fe^2+^ and Cu^+^, H_2_O_2_ is catalyzed to produce highly reactive hydroxyl radicals (OH), which can cause indiscriminate damage to nucleic acids, proteins, biofilms, and lipid peroxidation. This leads to mitochondrial depolarization, swelling, apoptosis, and cell death (17). Damaged and necrotic cells can activate Toll-like receptor 4 (TLR4) through damage-related molecular pattern (DAMP) activation. This leads to the aggregation of immune cells, which in turn express NADPH oxidase to promote the production of reactive oxygen species and further exacerbate myocardial damage (23). Overall, the overproduction ROS during I/R injury, along with the activation of TLR4 and NADPH oxidase, creates a vicious cycle that intensifies the damage to the myocardium.

### 2.2 Endothelial dysfunction

Endothelium regulates vascular tone, cell adhesion, thromboresistance, smooth muscle cell proliferation, and vascular wall inflammation by producing and releasing vasoactive molecules. This produces and releases vasoactive molecules such as prostaglandins, nitric oxide (NO), endothelium-dependent hyperpolarizing factors, and endothelium-derived contracting factors (24), which impact vascular tone, cell adhesion, thromboresistance, smooth muscle cell proliferation, and vascular wall inflammation. These molecules help to regulate the degree of vasodilation/contraction, tissue oxygen consumption balance, long-term organ perfusions, vascular structure remodeling, and metabolism (25). The integrity of the endothelial barrier depends on the intercellular junction complex located between adjacent endothelial cells (26). Endothelial dysfunction, characterized by impaired endothelial function, is primarily driven by oxidative stress and inflammation (27). Evidence has shown that endothelial injury is a key mediator of myocardial ischemia/reperfusion injury (26, 28). Additionally, I/R injury itself can lead to endothelial dysfunction, manifested by decreased nitric oxide production, vascular dystonia due to endothelial injury, and prolonged vasoconstriction.

During myocardial I/R, there is disruption of endothelial integrity and decreased microvascular permeability of cardiac myocardium after myocardial I/R (29), which increases the permeability of the endothelial barrier by destroying endothelial barrier function and aggravating the inflammation (30, 31). No reflux phenomenon of myocardium is also seen after I/R, leading to vascular leakage and neutrophil infiltration, and eventually apoptosis of cardiomyocytes and damage to myocardial function (30, 31).

### 2.3 Mitochondrial dysfunction

Mitochondria play a crucial role in oxidative stress and cell metabolism, and they are involved in various physiological functions such as endothelial mobilization, aging, proliferation, and growth (32). In cardiomyocytes, mitochondria are responsible for synthesizing about 90% of ATP, which is essential for normal heart functioning or cardiac functional recovery after various injuries (33, 34). Many studies have identified mitochondrial dysfunction as an important prominent mechanism for the progression of myocardial ischemia-reperfusion injury (34, 35). Abnormal mitochondrial fission, decreased mitophagy, and excessive mitochondrial oxidative stress can lead to endothelial dysfunction or death during cardiac reperfusion episodes (36). Mitochondrial dysfunction can lead to cell death through calcium imbalance, overproduction of mitochondrial ROS (mROS), disruption of cellular energy metabolism, impaired ATP production, and the opening of a structure called the mitochondrial permeability transition pore (MPTP), which can ultimately lead to cell death (34, 37). To counteract the effects of ROS and oxidative stress, mitochondria have a complex network of clearance systems (38). Studies have shown that abnormal mitochondrial fission can be an early indicator of mitochondrial dysfunction, and an imbalance between mitochondrial fission and fusion can lead to mitochondrial dysfunction, which in turn can aggravate MIRI damage (28, 34). Another factor contributing to injury is the accumulation of mitochondrial succinate, a metabolite that increases during hypoxia (39). This accumulated succinate is oxidized during reperfusion, resulting in the generation of ROS through a process called reverse electron transport (39). This excessive ROS production further contributes to oxidative stress and tissue damage. During myocardial ischemia, prolonged ischemia induces an increase in mitochondrial fission (40–42). When reperfusion occurs, the uncontrolled production of ROS triggers mitochondrial fission (34, 43, 44). This increased mitochondrial fission reduces mitochondrial membrane potential (MMP), making the MPTP more sensitive and leading to further ROS production. This disruption of the antioxidant balance within the mitochondria can result in the releasing of Cytochrome C (Cyt C) during cardiac microvascular I/R damage, activating caspases and initiating apoptosis through mitochondria-dependent pathways (45–48).

### 2.4 Calcium overload

Calcium plays an important role as the second messenger in various cellular processes, including cell proliferation, division, and energy metabolism. However, excessive calcium levels, known as calcium overload, can lead to detrimental effects in cellular function, as proposed by Zimmerman and Hulsmann in 1966 (49). In a stable internal environment, calcium inflow and outflow are dynamically balanced under the regulation of protein channels (50). Maintaining intracellular calcium homeostasis is crucial for the normal function and growth of cardiomyocytes. Calcium overload in cardiomyocytes can exacerbate ischemic damage, which occurs when blood supply to the heart is compromised (51). When calcium overload occurs, Ca^2+^ dependent protease can promote the conversion of xanthine dehydrogenase to xanthine oxidase, promote the production of reactive oxygen species, and the high concentration of Ca2+ in the cytoplasm increases mitochondrial uptake of Ca2+, which in turn forms calcium phosphate deposition in the mitochondria, and subsequently adversely affects ATP synthesis. Calcium homeostasis cannot be maintained during myocardial ischemia-reperfusion and intracellular calcium overload is a common pathway for irreversible damage of cells subjected to myocardial ischemia-reperfusion (52). During myocardial ischemia, adenosine triphosphate (ATP) production decreases, leading to intracellular acidosis and the activation of Na+/H+ exchange causing a large influx of sodium ions. This sodium influx, coupled with the high calcium concentration, contributes to calcium overload during reperfusion (53). Reperfusion also disrupts mitochondrial membrane potential and accelerates energy expenditure, resulting in mitochondrial calcium overload and excessive production of ROS. The accumulation of calcium ions in cells inhibits mitochondrial ATP synthesis, leading to instability in mitochondrial membrane potential and subsequent damage, such as contraction disorders and apoptosis (54), exacerbating post-hypoxic or post-ischemic cardiomyocytes injuries (40, 55, 56).

### 2.5 Endoplasmic reticulum stress

**Figure 1:**
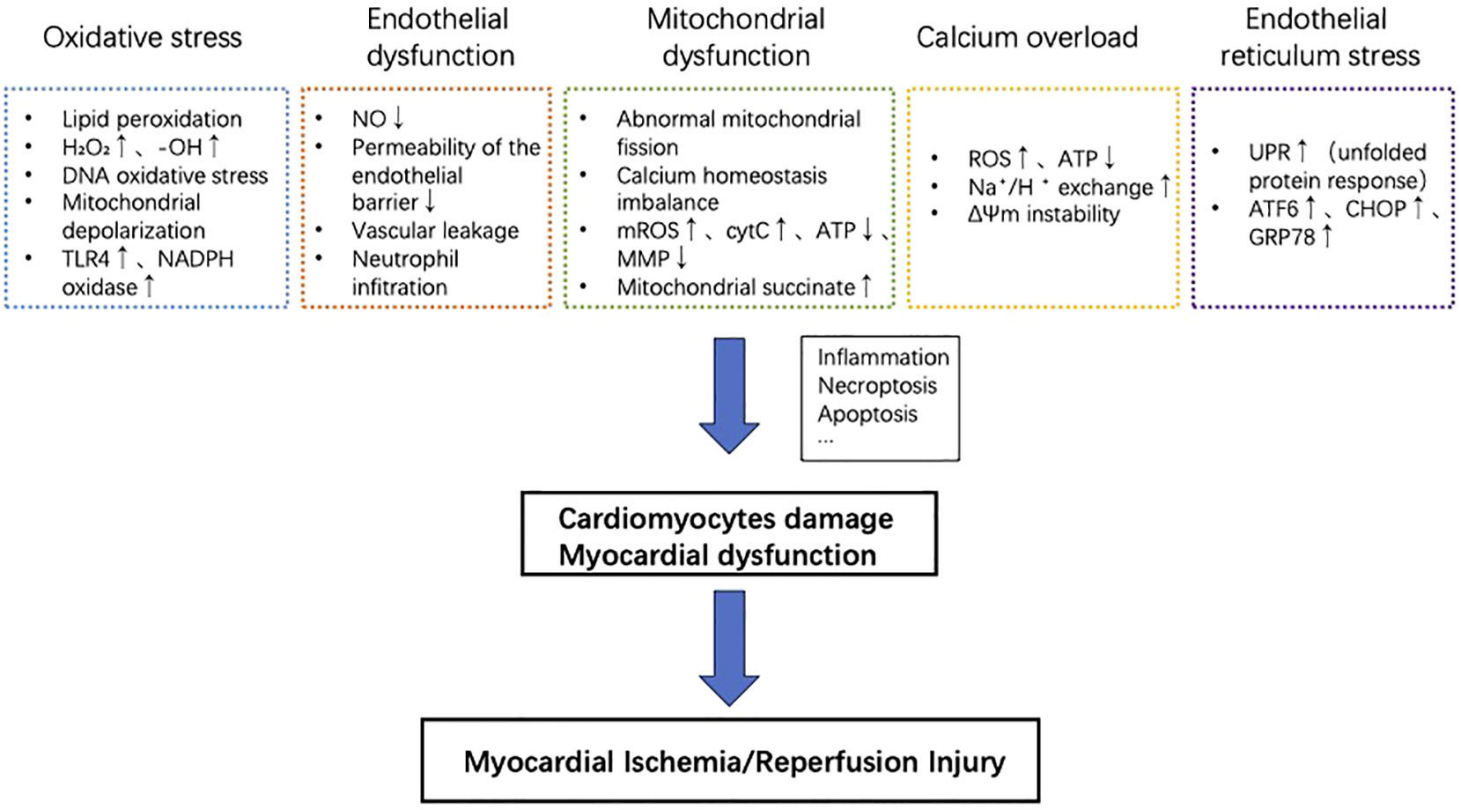
Key cellular events in the pathogenesis of MIRI. Oxidative stress, endothelial dysfunction, mitochondrial dysfunction, calcium overload, and reperfusion injury contribution to myocardial cell damage and cardiac dysfunction. These processes promote inflammation, necrotic apoptosis, apoptosis, and other mechanisms, ultimately exacerbating myocardial ischemia/reperfusion injury.

The endoplasmic reticulum (ER) regulates the synthesis, folding, and transport of a significant portion of proteins in eukaryotic cells (57). High-quality protein folding can determine cell survival and function as well as normal physiological function. Endoplasmic reticulum homeostasis involves the binding of three ER transmembrane proteins: protein kinase R-like ER kinase (PERK), activated transcription factor 6 (ATF6), and enzyme 1 (IRE1) (58). Such a binding keeps them inactive, and their activation requires specific conditions such as the presence of inositol (58). During environmental injury or disease state, the disruption of endoplasmic reticulum homeostasis can lead to protein misfolding and accumulation of unfolded proteins. This triggers a response called endoplasmic reticulum stress, which activates the unfolded protein response (UPR). The UPR is a cellular mechanism aimed at reducing the burden and damage caused by the ER stress. It helps to restore protein homeostasis within the ER and rebuild the endoplasmic reticulum balance. However, if the endoplasmic reticulum stress becomes chronic or severe, it can promote cell death. Myocardial ischemia is an example that induces ER stress response (59). Activation of the endoplasmic reticulum stress-related pathway induces downstream activation of the apoptotic pathway, thereby promoting the progression of ischemia/reperfusion injury in myocardial tissue (60). Cardiomyocytes express high levels of endoplasmic reticulum stress-related signaling proteins, including transcription factor 6 (ATF6), C/EBP homologous protein (CHOP), glucose regulatory protein 78 (GRP78), etc. Treatments that Inhibit the signaling of endoplasmic reticulum stress can effectively reduce the rate of cell death in conditions like myocardial ischemia-reperfusion injury (61–64). Key cellular events in the pathogenesis of MIRI are summarized in **Figure 1**.

### 2.6 Current therapeutic interventions against MIRI and the pro-survival cardiac protective signaling pathways

Ischemic heart disease (IHD) is one of the most common diseases that affects the human lifespan. Percutaneous coronary angioplasty, coronary artery bypass grafting, and other reperfusion methods such as thrombolysis treatment are the most effective treatment methods for myocardial ischemic injury up to date. However, post-ischemic reperfusion itself can cause new heart damage called “ischemia/reperfusion injury”. Although a variety of reperfusion therapies have matured, the development of therapies to reduce ischemia/reperfusion injury has been slow. Modern medical treatment has solved the technical problems of myocardial ischemia injury and blood flow recovery, but a series of complex processes of intracellular environmental changes that are caused by reperfusion after blood flow recovery have not been solved. The endogenous adaptive mechanism that occurs in cardiomyocytes in the face of ischemia-reperfusion or other types of metabolic stress challenges is called the pro-survival cardioprotective mechanism (65). The SAFE pathway, also known as the Survivor Activating Factor Enhancement pathway, is a signaling pathway that plays a crucial role in I/RI. This pathway is initially identified in studies investigating the cardioprotective effects of erythropoietin (EPO) against I/R injury (66, 67). It has been found that EPO activates the Janus Kinase (JAK) and signal transducer and activator of transcription (STAT) signaling pathway, particularly the JAK2/STAT3 pathway, to confer cardioprotection (68). In addition to EPO, other factors and pathways have also been implicated in activating the SAFE pathway. These include cytokines (such as interleukin-6 and interleukin-10), growth factors, and pharmacological agents (such as statins and opioids) (69). These factors can activate JAK/STAT signaling and trigger the downstream protective effects mediated by the SAFE pathway. The Reperfusion Injury Salvage Kinase (RISK) pathway is another signaling pathway that has a cardioprotective effect against I/R injury. It was first identified in studies investigating the cardioprotective effects of Ischemic Preconditioning (IPC), a phenomenon in which brief episodes of ischemia followed by reperfusion protect the heart against subsequent sustained I/R injury. Another cardioprotection mechanism that is relevant to this review is called postconditioning, which involves applying brief episodes of ischemia and reperfusion at the onset of reperfusion after a prolonged period of ischemia. This technique interrupts the initial reperfusion phase to protect the heart against I/R injury. The RISK pathway involves the activation of multiple protein kinases, including phosphatidylinositol 3-kinase (PI3K), protein kinase B (Akt), and extracellular signal-regulated kinase (ERK) (69–71). Activation of these kinases leads to the phosphorylation and activation of various downstream targets that confer cardioprotection. In addition to the aforementioned pro-survival protective signalings, intracellular signaling molecules are also involved in cardioprotective signaling pathways, such as protein kinase C (PKC), protein kinase A (PKA), protein kinase G (PKG), 5’ amp activated protein kinase (AMPK), p38 mitogen-activated protein kinase (MAPK), extracellular signaling regulatory kinase 1/2 (ERK1/2) (65, 72, 73). However, the interaction between them has not been fully determined (65, 72, 73).

## 3 Increased myocardial susceptibility to MIRI in diabetes

Diabetes is a major risk factor for IHD. Diabetes not only increases the incidence of acute myocardial infarction and myocardial sensitivity to ischemia-reperfusion injury but also alters or diminishes the myocardial response to cardioprotective interventions such as ischemic conditioning that are otherwise effective in subjects without diabetes. In animal models, ischemia preconditioning has been shown to be cardioprotective and reduce myocardial I/R damage. A recent study conducted in the db/db mouse model of type 2 diabetes shows that diabetes disturbs functional adaptation of the non-ischemic remote myocardium after ischemia/reperfusion (74). However, the effects of pretreatment-mediated cardioprotective in diabetic animal models are still controversial and inconclusive. In the study of Tatsumi et al., diabetic myocardial pretreatment stimulation produces a more substantial protective effect compared to regular myocardial pretreatment stimulation (75), while other studies have shown that diabetes attenuates or inhibits pretreatment-mediated cardioprotective effects (76). Diabetes can trigger various histological, biochemical, and physiological changes that contribute to the aggravation of oxidative stress, apoptosis, inflammation, and other pathways by increasing inflammatory factors, which leads to cardiac dysfunction, and exacerbating myocardial ischemia-reperfusion phenomenon (77).

### 3.1 High glucose induced increase in ROS in the heart

**Figure 2:**
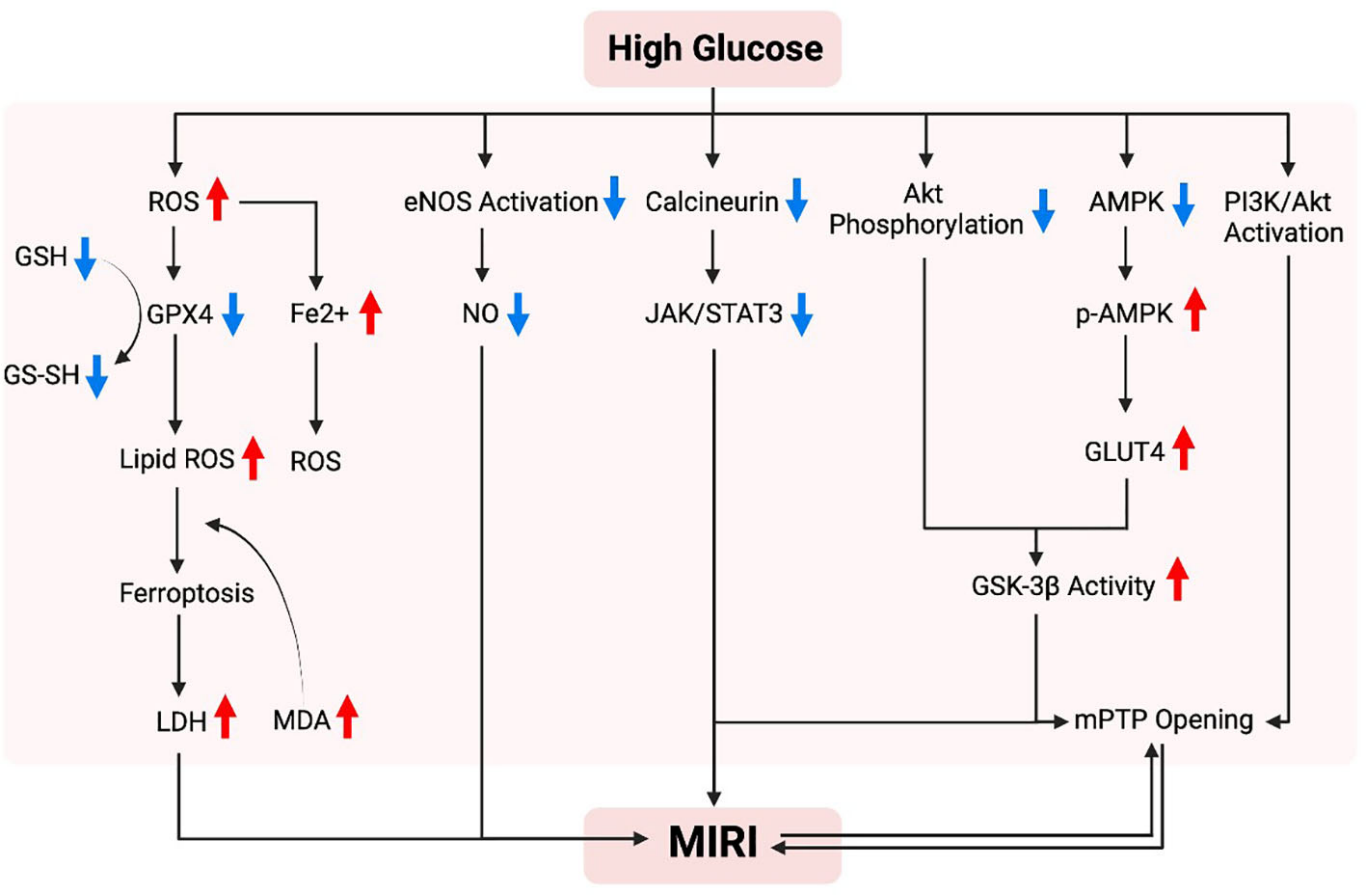
Possible mechanism of hyperglycemia in promoting myocardial ischemia-reperfusion injury. In a high-glycemic environment, there is an increased vulnerability to myocardial ischemia/reperfusion injury, which can be attributed to several mechanisms. These mechanisms include the overproduction of reactive oxygen species (ROS), an excessive burden of oxidative stress, abnormal alterations in mitochondria resulting in cell death due to iron overload, and dysfunction of endothelial nitric oxide synthase (eNOS). Furthermore, impaired protection against such injury is often linked to inadequate activation of pro-survival signaling pathways, including Akt, AMPK, JAK/STAT3, and PI3K/Akt.

**Figure 2** studies conducted in diabetic rodents indicate that high glucose enhance superoxide generation and mitochondrial structural changes that increase the vulnerability of the myocardium to IR injury (78), and treatments that have anti-oxidant property attenuate myocardial IRI through improving mitochondrial homeostasis (28). Thus, excessive oxidative stress and impaired mitochondrial biogenesis in diabetic conditions rendered the diabetic heart more vulnerable to ischemic insults (56, 79). Mechanistically, Nrf2 nuclear translocation triggers Sirt3 upregulation and MnSOD activation, which subsequently reduces mitochondrial levels of ROS (mtROS). However, high glucose levels cause a downregulation of Nrf2 levels in the nucleus, resulting in Sirt3 downregulation and the acetylation of manganese superoxide dismutase (MnSOD), and thus facilitating the production of ROS (80). Energetic stress and mitochondrial ROS formation play critical roles in the pathogenesis of diabetic cardiomyopathy and MIRI (81, 82). AMPK, an important kinase involved in regulating energy homeostasis, plays a role in various metabolic process such as protein metabolism, lipid metabolism, carbohydrate metabolism, autophagy, and mitochondrial homeostasis. It is known that AMPK can sense cellular metabolic conditions and promote mitochondrial biogenesis. In the absence of glucose and ATP, AMPK is activated. Activation of AMPK has been shown to reduce the production of ROS and protect mitochondrial biogenesis. In the presence of high glucose, there is a dual inhibitory effect on AMPK. High glucose reduces the protein level and kinase activity of AMPKα, the catalytic subunit of AMPK. Researchers discovered that high glucose stimulation did not cause an increase in ATP levels, but it did cause an increase in the ratio of AMP/ATP and ADP/ATP (83). This suggests that ATP is not the cause of high glucose inhibition of AMPK signaling. Instead, high glucose promotes the production of ROS in cells (83). Under conditions of persistent hyperglycemia, elevated ROS levels are a causative factor in cell death (84). Under normal physiological conditions, cells have an antioxidant system to remove excess ROS. However, in diabetes, there is a decrease in antioxidant system activity and an increase in ROS production. This impairment between oxidant and antioxidant systems lead to oxidative stress, and results in various forms of damage to cells and tissue (84).

### 3.2 High glucose induced ferroptosis

Iron death, also known as ferroptosis, is a process in which iron-dependent cell death occurs due to increased lipid peroxidation. It is involved in various pathological processes such as cancer drug resistance, neurodegenerative diseases, IR/I and more (85). Several factors and markers are associated with iron death, including lactate dehydrogenase (LDH) activity, lipid peroxidation by reactive oxygen species (ROS), iron (Fe2+) levels, glutathione (GSH) levels, and malondialdehyde (MDA) levels. Studies have shown that high glucose (HG) conditions can increase ROS production with subsequently increased production of the lipid peroxidation product MDA, which in the presences of increased Fe2+ levels but decreased GSH and GPX4 levels, jointly lead to the induction of iron death (86). HG intake also leads to increased production of ROS, exacerbating oxidative stress increasing the production of GSSG, and reducing GSH content (87). This can result in mitochondrial abnormalities, such as decreased size, loss of mitochondrial ridges, and damage to the outer mitochondrial membrane. Glutathione peroxidase 4 (GPX4) is a key regulator of iron death, and its protein levels are significantly reduced under HG condition. The reduction in GPX4 leads to increased lipid ROS formation, lipid peroxidation, and ultimately cell iron death (86). Studies have found that adding ferroptosis cell death inhibitor can reduce cell death in HG environments, indicating the potential therapeutic importance of targeting this pathway (85). Overall, the process of ferroptosis and the proteins associated with it are strongly linked to glucose and lipid metabolism disorders (88). Herb extracts that antioxidant and anti-inflammatory properties such as Astragaloside IV has been shown to attenuate diabetic heart dysfunction in rats via inhibiting ferroptosis (89). However, studies regarding the relative role of ferroptosis in the diabetic myocardial IRI are rare and not definitive (77, 90, 91).

### 3.3 Impaired signaling such as eNOS, STAT3, PI3K/Akt in diabetes

Endothelial nitric oxide synthase (eNOS) is an enzyme that produces nitric oxide (NO) in the endothelial cells of blood vessels. NO is the key regulator of vascular function and homeostasis, and it plays a crucial role in maintaining the health of the cardiovascular system. Dysfunction of eNOS has been found to be associated with the development of diabetes (92). Studies have shown that eNOS dysfunction is closely linked to a high glucose environment, which is characteristic of diabetes. Restoring normal eNOS function is essential for improving vascular health in individuals with diabetes (93). In fact, upregulation of eNOS expression has been found to have a protective effect in diabetic patients (94). It has been discovered that eNOS uncoupling, which is the loss of balance between NO production and ROS generation, is a significant source of increased ROS production in diabetes. Increased oxidative stress further exacerbates eNOS uncoupling and endothelial dysfunction, contributing to cardiovascular damage (95). The PI3K/Akt/eNOS signaling pathway is also affected/impaired by diabetes (96, 97). This pathway involves phosphatidylinositol 3-kinase (PI3K) and AKT/protein kinase B (PKB/AKT), which respond to external signals and regulate various cellular processes, including metabolism, proliferation, cell survival, growth, and angiogenesis (98). Impairment in this pathway can lead to both cardiovascular damages in diabetes (79, 99). The activation of the PI3K-AKT pathway has been shown to play a crucial role in protecting the heart from myocardial IR/I. Cardiac protective interventions such as Ischemic preconditioning, a process that exposes the tissue to brief periods of ischemia before a more prolonged ischemic event, can activate the PI3K-AKT pathway and provide protection to the heart (73, 100). Additionally, the Akt and JAK/STAT3 signaling pathways have been found to be involved in reducing diabetic heart I/R damage (101). However, both the PI3K-AKT pathway and the JAK/STAT3 signaling pathway are impaired in the myocardium of diabetic subjects (102, 103), rendering the diabetic hearts more vulnerable to ischemia reperfusion injury and less or not sensitive to therapeutic interventions that are otherwise effective in non-diabetic subjects (104–106).

## 4 Cardioprotective effects of salvianolic acid A and salvianolic acid B against MIRI

Salvianolic acid is a compound found in the herb salvia, and it has been found to have several beneficial properties, including antioxidant, anti-inflammatory, and antiplatelet properties (107). In the context of myocardial IR/I, salvianolic acid has shown potential cardioprotective effect. Studies have indicated that salvianolic acids, specifically SAA and SAB, can help reduce damage to cardiomyocytes during MIRI in a rat model of ischemia-reperfusion injury (108). However, the exact mechanism by which salvianolic acid exerts its protective effects on MIRI is still not fully understood.

### 4.1 Salvianolic acid A

**Figure 3:**
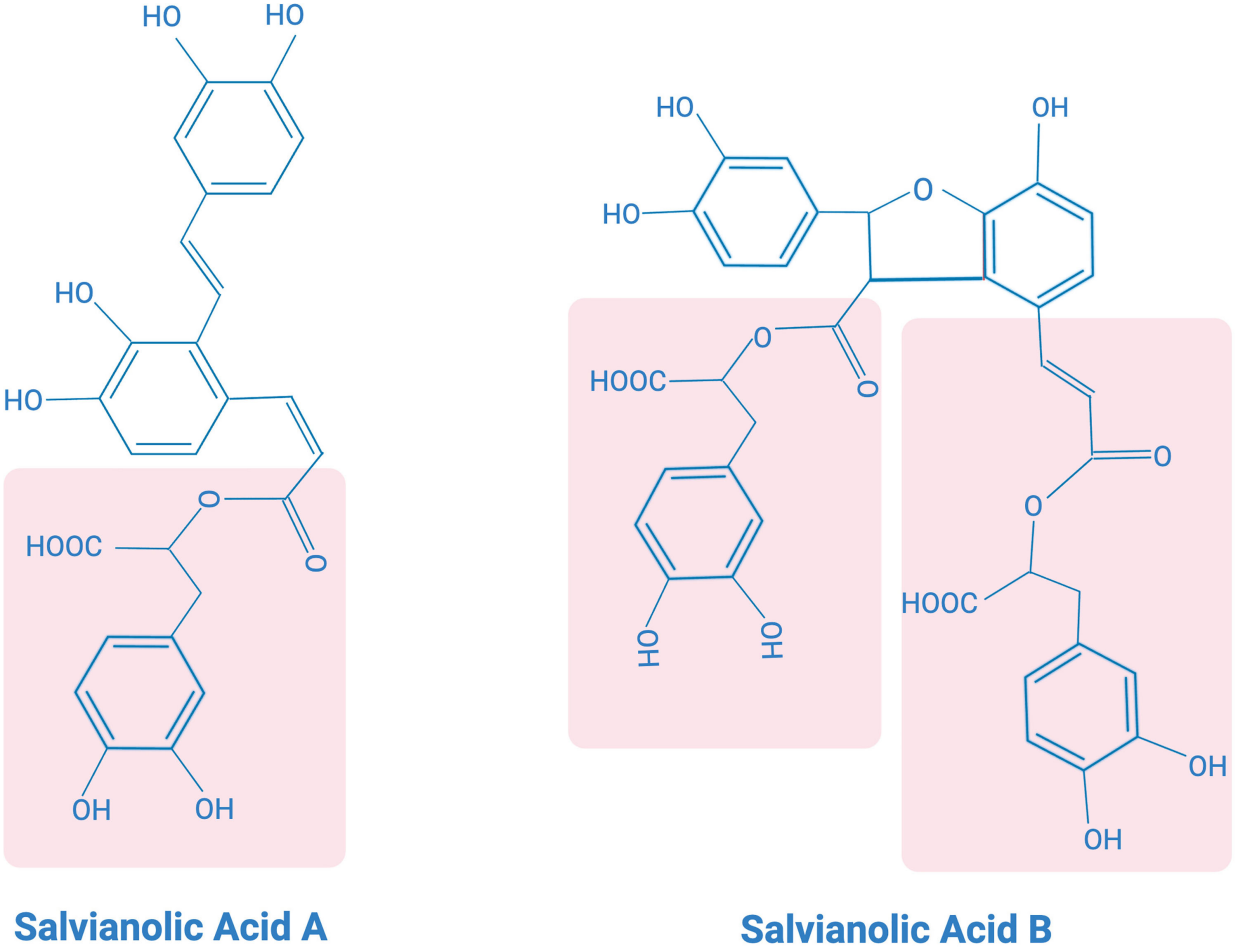
Chemical structures of salvianolic acid **(A)** and salvianolic acid **(B)**.

SAA possesses a polyphenolic acid chemical structure (**Figure 3**), exhibiting strong antioxidant capacity. It has also been found to have antioxidant, anticancer, antifibrotic, anti-inflammatory and antiplatelet aggregation properties (109). *In vitro* studies have shown that SAA exhibits potent free radical scavenging ability assessed by the methods of 1,1-diphenyl-2-picrylhydrazyl (DPPH) radical scavenging assay and 2,2-azino-bis-(3-ethylbenzothiazoline-6-sulfonic acid (ABTS(+)) radical cation decolorization assay (110). SAA has been shown to attenuate myocardial functional impairment and cell death caused by oxidative stress and attenuate hydrogen peroxide-induced oxidative stress damage to cells both *in vivo* in rodent models and *in vitro* in cultured H9c2 cardiomycytes (111, 112). Experimental evidence has demonstrated that SAA pretreatment upregulates the anti-apoptotic protein Bcl-2 and inhibits pro-apoptotic proteins Bak and Bax, thereby inhibiting apoptosis (113). Additionally, SAA has been found to significantly ameliorate mitochondrial dysfunction caused by myocardial ischemia in an isoprenaline-induced myocardial ischemia a rat model (114). The contractile function of cardiomyocytes, as reflected by their shortening, was dose-dependently improved by SAA after myocardial ischemia-reperfusion (115). Furthermore, SAA pretreatment has been shown to reduce lactate dehydrogenase (LDH) leakage in ischemic myocardium, decrease LDH release from the ex vivo heart, and significantly improve cell viability. SAA also downregulates the expression of cleaved caspase-3 protein, thereby inhibiting apoptosis in cardiomyocytes. These findings suggest that SAA pretreatment before ischemia-reperfusion inhibits cardiomyocytes necrosis and apoptosis, thereby reducing ischemia-reperfusion-induced cardiomyocyte damage (115, 116).

### 4.2 Salvianolic acid B

SAB has been shown to effectively attenuate cardiovascular injuries by reducing the expression of related inflammatory factors, inhibiting apoptosis, and reducing oxidative stress in experimental settings (117). Numerous studies have demonstrated the cardiomyocyte protective effects of SAB during myocardial ischemia-reperfusion injury (MIRI) and its ability to reduce oxidative stress-induced damage (118). Similar to SAA, SAB also reduces post-ischemic LDH leakage (119). Experiments investigating the cardioprotective effect of SAB on myocardial ischemia-reperfusion injury, based on cell viability and LDH leakage, have shown that SAB inhibit autophagy, enhances cell viability, reduce LDH leakage, and increases the survival rate of cardiomyocytes after I/R (120). There is evidence showing that the cardioprotective effect of SAB on MIRI is dose-dependent, and both high and low doses of SAB have been found to reduce the size of myocardial infarction after treatment. Moreover, SAB effectively reduces cardiomyocyte apoptosis by significantly increasing the ratio of Bcl-2 expression to Bcl-2/Bax and reducing Bax expression (121). During ischemia-reperfusion, a large amount of ROS is released, accompanied with increased lipid peroxidation product malondialdehyde (MDA) (122) and other specific indicators of ROS-induced lipid peroxidation such as 15-F2t-Isoprostane (123, 124). Studies have demonstrated that SAB treatment can reduce high levels of malondialdehyde measured in rat models of testicular ischemia-reperfusion and myocardial ischemia-reperfusion, with no significant side effects observed throughout the treatment (108, 125). Recent research has also shown that SBB attenuates post-ischemic myocardial apoptosis, inhibits ROS production, decreases MDA levels, and enhances superoxide dismutase (SOD) activity through a mechanism that involves the regulation of the TRIM8/GPX1 axis *in vivo*, making it a potential candidate for the prevention or treatment of MIRI in cultured AC16 cardiomyocytes (118).

### 4.3 Impacts of salvianolic acid A and salvianolic acid B on the signaling pathways affecting MIRI

**Figure 4:**
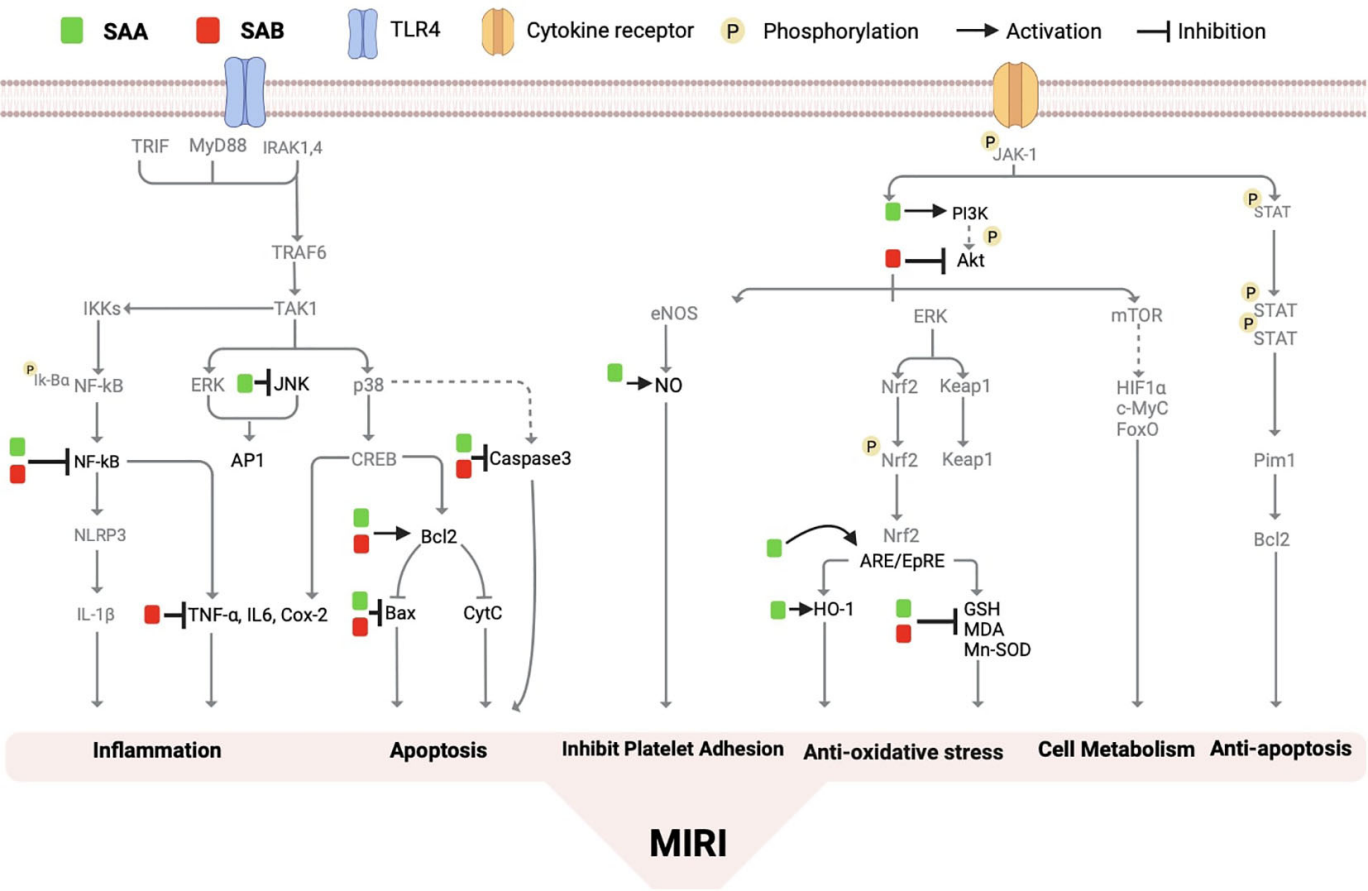
The molecular mechanism of salvianolic acid A and salvianolic acid B on myocardial ischemia/reperfusion injury. Salvianolic acid A and salvianolic acid B have the ability to regulate various molecular pathways including nuclear factor kB (NF-kB), stress-activated protein kinase (Bax, Caspase 3, malondialdehyde (MDA), heme oxygenase-1 (HO-1), bcl2, phosphatidylinositol 3-kinase (PI3K), nitric oxide (NO), antioxidant reaction element (ARE), tumor necrosis factor (TNalpha), protein kinase B (PKB/Akt), interleukin-6 (IL-6), and others. By inhibiting the production of reactive oxygen species, these compounds effectively improve inflammation, apoptosis, autophagy, microcirculation disorders, cell growth, and metabolism.

In rat models of myocardial IRI, Salvianolic Acid A (SAA) pretreatment has been shown to significantly reduce post-ischemic myocardial infarction concomitant with reduced plasma levels of cTnT, CK-MB, TNF-α and IL-1β compared with untreated I/R group (126). Platelets play a critical role in I/R injury, as activated platelets produce various factors that promote blood clot formation. It has been found that SAA treatment can resist ADP and collagen-induced human blood platelet aggregation and thrombosis by inhibiting the abnormal increase of the phosphorylation of Akt and also inhibits PI3K, and these effects of SAA was comparable to that of the PI3K inhibitor LY294002 both *in vitro* and *in vivo* in a mouse model of arterial thrombosis (127). NO is known to play a central role in maintaining cardiovascular homeostasis, and SAA treatment increases rat left ventricle NO content after myocardial I/R (126). Studies have found that SAA can also exerts cardioprotective effects through the ERK1/2 pathway, and this effect is inhibited by the ERK2/098059 inhibitor PD600125 (PD), suggesting that the protective effect of SAA on I/R cardiomyocytes may also depend on the inhibition of the JNK pathway (128). SAA has also been found to inhibit I/R-induced cardiomyocyte apoptosis through the PI3K/Akt, JNK, and ERK1/2 pathways. Among these pathways, ERK1/2 and JNK are regulated by upstream kinases (MAPK kinases), which activate each other in a stepwise manner. Experimental results have shown that inhibition of the p38 MAPK signaling pathway and the JNK signaling pathway can effectively protect and improve MIRI (128). Additionally, Salvianolic Acid B (SAB) has been shown to regulate the PI3K/Akt pathway, and to inhibit apoptosis by downregulating JNK phosphorylation, BCL2-associated X (Bax)/B-cell lymphoma-2 (Bcl-2), and caspase-3 expression (121) In addition, a recent study demonstrated that ubiquitin-proteasome degradation of GPX4 occurs in both MIRI models in rats and in *in vitro* models of cardiomyocyte hypoxia/reoxygenation, while SAB can reduce this degradation and inhibit ferroptosis and apoptosis of cardiomyocytes during MIRI and H/R and protect the cardiovascular system by the GPX4/ROS/JNK-mediated crosstalk mechanism (129). SAA and SAB have respectively been demonstrated to regulate the Jak/STAT3 signaling pathway in the liver (130) and in the intervertebral discs in rats (130). However, the potential impacts of SAA and SAB on the Jak/STAT3 signaling in the heart especially in the context of MIRI have not been explored thus far, which merits in depth future study given the critical role Jak/STAT3 signaling pathway plays during MIRI (45, 131, 132). The current understandings regarding the impacts of SAA and SAB on the signaling pathways that affect MIRI are summarized respectively in **Figure 4**.

## 5 Cardioprotective potential of salvianolic acid A and salvianolic acid B against MIRI in diabetes

In this study, we provide a systematic summary of the cardioprotective mechanism of salvianolic acid in diabetic myocardial ischemia-reperfusion injury, as well as various signaling molecules and mechanisms associated with myocardial I/R injury. Both salvianolic acid A and B have the potential to exert cardioprotective effects, either through similar or different mechanisms (**Table 1**). The relevant signaling pathways involved in myocardial ischemia-reperfusion injury include phosphatidylinositol-3 kinase/Akt (PI3K/Akt), mitogen-activated protein kinases (MAPKs), JANUS kinase/signal transduction and transcriptional activators (JAK/STAT), nuclear factor-κB (NF-κB), and others (96). Studies have revealed that diabetes can further impair the phosphatidylinositol 3-kinase/Akt/eNOS (PI3K/Akt/eNOS) pathway and activate JAK/STAT3 signaling, thereby exacerbating myocardial ischemia-reperfusion injury in diabetic rats which can be attenuated by treatment with SAA (96).

**Table 1:** Salvianolic Acid potential cardiomyocytes in diabetic myocardial ischemia/reperfusion injury.

	Cell Line	Animal	Mechanism	Effect Factors	Reference	Year
**Salvianolic acid A**	H9C2	Mouse	TRL4↓、MyD8↓ p-JNK↓、p-ERK1/2↓	anti-inflammatory mitochondrial dysfunction↑	(133)	2021
	–	Rat	Bcl-2↑、Bax↓ JNK / PI3K / Akt	anti-apoptosis LDH leakage↓ infarct size↓	(96)	2016
	–	–	PI3K↓Rap 1↓ late PI3K-dependent Akt phosphorylation↓	platelet adhesio↓ platelet activati↓	(127)	2010
	HK-2	Rat	MDA↓、HUVECs↓ VCAM-1↓、HO-1↑ Nrf2↑	oxidative stress↓ inflammation↓	(134)	2016
	HepG2	Mouse Rat	ATP↑ MMP↓ CaMKKβ / AMPK↑	myocardial dysfunction↑	(135)	2015
	–	Rat	NF-κB ↓	anti-inflammatory anti-apoptosis	(109)	2022
	–	Rat	AST↓、CK↓ LDH↓	anti-inflammatory	(114)	2009
	–	Rat	Bcl-2↑、Bax↓ Bcl-2/Bax↑ Caspase-3↓	anti-apoptosis anti-necrosis	(115)	2011
	H9C2	Rat	SOD↑、Bcl2↑ H2O2-↓、p-Erk1/2↓	infarct size↓ anti-apoptosis	(136)	2011
	–	Rat	TNF-α↓、IL-1β↓ NO↑	anti-inflammatory myocardial dysfunction↑ platelet aggregation↓ anti-platelet	(126)	2017
**Salvianolic acid B**	H9C2	–	NF-κB ↓、IL-6↓ IL-1β↓、TNF-α↓	anti-inflammatory anti-apoptosis	(137)	2016
	ESC	–	HIF1α↓ 、BNIP3↓ cleavage caspase 3 ↓	anti-apoptosis	(138)	2015
	H9C2	–	ΔΨm↑、caspase-3↓ LC3-II↓	Anti-mitochondrial auto-phagy	(139)	2020
	–	Rat	P-Akt↑、HMGB1↓ TLR4↓	infarct size↓ anti-apoptosis	(121)	2019
	INS-1	–	caspase-9↓ caspase-3↓ MDA↓	anti-apoptosis	(140)	2017
	–	–	–	inhibits platelet adhesion	(141)	2008
	HUVEC	Rat	VEGFR2↑、VEGFA↑ IGFBP3↓、p-Akt↑	ameliorated left ventricular dysfunction and remodeling cell proliferation↑	(142)	2020
	–	Rat	SIRT1↑、Bcl-2↑ Ac-FOXO1↓、Bax↓	anti-inflammatory anti-apoptosis	(117)	2015
	AC16	Rat	TRIM8/GPX1	oxidative stress↓ anti-apoptosis	(118)	2022
	primary myocardial cells	–	miR-30a↑、LDH↓ PI3K / Akt	anti-autophagy	(120)	2016
	–	Rat	P-Akt↑ HMGB1↓	infarct size↓ anti-inflammatory myocardial dysfunction↑ anti-apoptosis	(121)	2019

“-” means no mention.

“↑” means increased or enhanced.

“↓” means decreased or reduced.

### 5.1 Salvianolic acid A and/or salvianolic acid B can reduce diabetic myocardial ischemia-reperfusion injury

As mentioned earlier, SAA pretreatment has been shown to have a protective effect on the myocardium during I/R in non-diabetic rats. Further studies have found that Sal A pretreatment significantly improved cardiac hemodynamic and reduced LDH activity after I/R in diabetic rats, with concomitant reduction in post-ischemic myocardial infarction apoptosis (96). Similarly, SAB has been found to significantly reduce intracellular reactive oxygen species and malondialdehyde (MDA) levels, effectively reduce oxidative stress induced by high glucose rat insulinoma cell line INS-1 cells (140). Clinical studies have observed that patients with antiplatelet therapy appears to have similar effects in patients with diabetic coronary artery disease compared to patients with non-diabetic myocardial ischemia-reperfusion injury (143). Sal B has been shown to inhibit platelet aggregation and platelet adhesion by interacting with collagen receptors (141). In subsequent studies, SAA has also been found to significantly inhibit agonist-induced platelet activation by inhibiting PI3K (127). The nuclear factor E2 related factor 2 (Nrf2)/heme oxygenase-1 (HO-1) signaling pathway is involved in the regulation of MIRI damage (144). Pro-inflammatory cytokines also play a significant role in diabetic vascular damage. VCAM-1, a pro-inflammatory cytokine, is known to be inhibited by Nrf2-mediated upregulation of HO-1 in vascular diseases. SAA has been found to reduce VCAM-1 expression by mediating the Nrf2/HO-1 signaling pathway (134).

### 5.2 Signaling pathway for possible cardiac protection of salvianolic acid A and salvianolic acid B in diabetic MIRI

Studies have demonstrated that insulin has cardioprotective effects mediated by the Akt signaling pathway, leading to the activation of eNOS through PI3K/Akt activation (145). The impairment of the PI3K/Akt signaling pathway is involved in myocardial I/R damage in diabetic rats, and high glucose further inhibits the PI3K/Akt pathway. Experimental evidence supports the significant increase in SERCA2 activity through JNK/PI3K/Akt signaling, resulting in anti-apoptotic effects and improvement in cardiac contraction and diastolic function in diabetic rats. Chen et al. found that SAA pretreatment significantly increased the level of the anti-apoptotic protein Bcl-2 in diabetic rats through the JNK/Akt signaling pathway, while reducing the levels of pro-apoptotic proteins Bax and cleaved-caspase-3, ultimately increasing the Bcl-2/Bax ratio and protecting against myocardial I/R damage in diabetic rats (96). Similarly, Sal B has been shown to reduce the expression of insulin-like growth factor binding protein 3 (IGFBP3) induced by high glucose, leading to the phosphorylation of extracellular signal-regulating protein kinase and protein kinase B (AKT) activity in rat models of diabetic cardiomyopathy and in cultured HUVECs under hypoxia (142). SAB is also considered a potent inhibitor of the Akt/mTOR pathway, reducing the phosphorylation of Akt and its downstream target mTOR (146, 147). Furthermore, Sal A can regulate glucose metabolism by increasing ATP production with concurrent reduction of mitochondrial membrane potential (MMP), and improving mitochondrial function through Ca^2+/^calmodulin-dependent protein kinase kinase-β (CaMKKβ)/AMPK signaling pathway in both type 1 and type 2 diabetic mice (135). NF-κB, which induces inflammatory factors involved in cardiomyocyte apoptosis, is also a downstream target of STAT3. Inhibition of NF-κB activity can prevent H9C2 cardiomyocyte apoptosis (148). Studies have found that SAB can inhibit the activation of the MAPK/NF-κB pathway induced by ox-LDL (149). SAB pretreatment has also been reported to reduce NF-κB levels (150), but whether SAB directly targets NF-κB or acts through its upstream pathway Akt/JAK or STAT3 remains unclear (113).

## 6 Conclusion

This review highlights the evidence and possible mechanisms by which salvianolic acid may reduce diabetic myocardial I/R damage. Possible mechanisms include modulation of oxidative stress, inflammatory response, mitochondrial dysfunction, ferroptosis and apoptosis through pathways such as PI3K/Akt, JAK/STAT, and NF-κB. A recent study has found that aldehyde dehydrogenase 2 (ALDH2) can activate the PI3K/AKT/mTOR pathway to alleviate ischemia and reperfusion injury in diabetic cardiomyopathy (44). However, it should be noted that studies have shown that Sal B can inhibit the Akt/mTOR pathway (147). There is currently no research showing that Sal B can exert cardioprotective effects by mediating the Akt/mTOR pathway. Although salvianolic acid has been studied in various clinical studies as an active ingredient in salvia, its research on diabetic myocardial I/R damage is relatively limited. Further understanding of the mechanisms underlying salvianolic acid-related myocardial protection will contribute to the development of new protective strategies and discovery of more effective therapies against diabetic myocardial I/R damage in the future.

## Author contributions

YJ: Writing – original draft. YC: Writing – original draft. RH: Writing – review & editing. YX: Supervision, Writing – review & editing. ZX: Supervision, Writing – review & editing. WX: Supervision, Writing – original draft, Writing – review & editing.
